# Skin-to-skin contact and delivery room practices: a longitudinal survey conducted in Piedmont and the Aosta Valley

**DOI:** 10.1186/s13052-019-0688-9

**Published:** 2019-08-02

**Authors:** Michelangelo Barbaglia, Enrico Finale, Silvia Noce, Alessandro Vigo, Cesare Arioni, Raffaella Visentin, Elisabetta Scurati-Manzoni, Andrea Guala, SUPC Piedmont and Aosta Valley study group, R. Artino, E. Audenino, L. Azzoni, G. Ballardini, R. Basta, A. Brach del Prevert, P. Capalbo, V. Castella, M. Colivicchi, G. Cosi, B. Cucchi, D. Farina, F. Ferrero, M. Frigerio, G. Galvagno, P. Gancia, O. Haitink, A. Marra, M. Nangeroni, A. Perona, M. Pescarmona, A. Serra, P. Serravalle, E. Uga, L. Vivalda, M. Zaffaroni

**Affiliations:** 1 Struttura Complessa Pediatria, Ospedale Castelli, Azienda Sanitaria Locale VCO, Verbania, Italy; 2 Centro di Riferimento Regionale per la SIDS, ASO/ OIRM/Sant’Anna, Torino, Italy; 3 Struttura Complessa Pediatria, Ospedale Umberto Parini, Azienda Sanitaria Locale della Valle d’Aosta, Aosta, Italy; 4 Dipartimento Materno Infantile VCO, Ospedale Castelli, Verbania, Italy

**Keywords:** Neonatal collapse, SUPC, Breast feeding, Delivery room practices, Skin to skin

## Abstract

**Background:**

Sudden unexpected postnatal collapse of presumably healthy neonates during early skin-to-skin contact is a rare, yet recognized occurrence, associated with a high risk of mortality and morbidity. A survey was conducted in 2012 in 30 delivery wards throughout Piedmont and the Aosta Valley to evaluate the environmental and logistical aspects that could be linked to SUPC. The survey was again conducted in 2016 in 28 delivery wards in Piedmont and the Aosta Valley in order to evaluate organizational improvements introduced after ministerial indications and recommendations by the Italian Society of Neonatology were published in 2014, in light of new findings regarding the phenomenon.

**Methods:**

A questionnaire specifically asking about the organization of delivery wards, and surveillance or supervision during early skin-to-skin contact, was sent to all of the hospitals taking part in the survey in both 2012 and 2016. The collected data were elaborated anonymously and the statistical analysis was performed by using the two by two table.

**Results:**

In 2012, 28 out of 30 delivery wards in Piedmont and Aosta, with a total of 31,074 newborns out of 35,435, were evaluated in all of the environmental and logistical aspects that might be cause for SUPC to occur. An identical survey was taken again in 2016; 26 out of 28 wards participated with a total of 27,484 newborns out of 30,339. In 2012, early skin-to-skin contact took place immediately in all the delivery rooms in 27 wards, and soon after in the post-partum room in one; in 11 out of 28 wards there was early skin-to-skin contact in the operating theater itself, following caesarean sections (11/26 in 2016). Routine newborn care was given after 3 h in 8 delivery wards (7/26 in 2016); after 2 h in 12 (7/26 in 2016); after 1 h in 2 (4/26 in 2016); after 30 min in 3 (2/26 in 2016); after 10 min in 1 (0/26 in 2016); after 1 or 2 min in 1 (0/26 in 2016) and at any time in one ward (6/26 in 2016).

**Conclusion:**

Periodic surveys of delivery wards are useful for the assessment of all the aspects and risk factors that need to be changed in order to implement safe early skin-to-skin contact.

**Electronic supplementary material:**

The online version of this article (10.1186/s13052-019-0688-9) contains supplementary material, which is available to authorized users.

## Introduction

Sudden Unexpected Postnatal Collapse (SUPC) of presumably healthy neonates within a few hours or days from birth is a rare yet recognized occurrence, associated with a high risk of mortality and morbidity (i.e. neurodisability) [5, 7, 10, 14]. According to the most recently published data, 36% of SUPC events occur during early skin-to-skin contact in the first 2 hours of life, mainly in the delivery room, while 53% of SUPC occur between the first 2 and 72 h of life in the delivery ward; 9% may still occur between 72 h and up to 7 days after birth, after mother and infant are discharged from hospital [8, 14]. Risk factors for SUPC have already been discussed and thoroughly investigated in previous studies, the most recognized of which are: primiparity; prone position of neonate; unsupervised skin-to-skin contact; initial breastfeeding during skin-to-skin contact; and analgesia and sedation of the mother [5, 7, 8, 14, 18]. In 2012, a survey was conducted in 30 Piedmont and Aosta delivery wards in order to evaluate all of the possible environmental and logistical aspects that could be addressed and changed so as to minimize the risk of SUPC occurring. A second survey, identical to the 2012 questionnaire, was conducted again in 28 delivery wards in 2016 in Piedmont and Aosta Valley to evaluate adhesion to the new ministerial indications and most recent findings published on the subject [8, 17].

## Methods

In the 2012 survey a questionnaire (Available as an Additional file 1) was sent via email to the head physicians of the 30 participating delivery wards, specifically asking about the organization of the ward, and surveillance or supervision, during early skin-to-skin contact. Each head physician completed the questionnaire, collaborating with the head midwife in the delivery room. Three months later the wards were contacted and asked to return the questionnaires, 28 of which were received correctly completed; the data were then collected and elaborated anonymously. The same questionnaire was sent out again in 2016 to the head physicians of the 28 participating wards, with their return requested 3 months later. The questionnaire is available in Italian as well as in English upon request. The collected data were elaborated anonymously and the statistical analysis was performed by using the two by two table.

## Results

In the 2012 survey, 28 out of 30 delivery wards in Piedmont and the Aosta Valley were evaluated regarding all of the environmental and logistical aspects that could represent grounds for SUPC to occur. Thirty-one thousand seventy-four neonates were involved then out of 35,435. An identical survey was conducted again in 2016; 26 out of 28 delivery wards replied with a total of 27,484 neonates out of 30,339.

The collected data have been grouped into three different sections:

1. **Early skin-to-skin contact: practice, timing and duration**

In 2012 early skin-to-skin contact took place in all of the delivery wards; immediately in the delivery room in 27 wards; and soon after birth in the post-partum room in one of them. In 2016 in all delivery wards skin-to-skin contact immediatly in delivery room. In 11 out of 28 delivery wards, and in 11 out of 26 in 2016 early skin-to-skin contact also took place in the operating theater after caesarean sections were performed.. In 12 (13 in 2016) delivery wards early skin-to-skin contact was suggested when the mother gave birth under sedation, but only under specific conditions: with the mother awake, and under closer or continuous observation, and after measures to insure bed safety were taken (*p* = 0.91;[95% CI 0.3995–3.013]). Contact was offered to fathers in 2 delivery wards both in 2012 and 2016. In 24 out of 28 in 2012 and in 26 out of 26 in 2016 delivery wards, early skin-to-skin contact began between 0 and 5 min after delivery (*p* = 0.84;[95% CI 0.5403–2.416]). In 2012 in one of the wards it occurred 5 min after delivery, in another 10 min after delivery, and 15 min after delivery in two other wards. In 18 delivery wards early skin-to-skin contact lasted as long as the mother wanted (15/26 in 2016) without specifying how much time in total; in 2 as long as the midwife decided, (3/26 in 2016); and in one ward it was left up to the pediatrician (0/26 in 2016). The answers “decide the midwife” and “decide the pediatrician” have been indicated as another reason of suspension to skin-to-skin. Mothers were also found in different positions during early skin-to-skin contact: in 13 delivery wards they were freely lying in the most comfortable position; in 6 they were lying in a supine position (8/26 in 2016); and partially sitting up in 8 (7/26 in 2016). Neonates were lying prone between the mother’s breasts in 24 delivery wards (20/26 in 2016). In all the delivery wards except one, neonates were covered with only their heads visible (0/26 in 2016). In 5 out of 28 delivery wards no non-medical staff was present during early skin-to- skin contact (2/26 in 2016), whereas in 23 out of 28 delivery wards either the father, other family members or close friends were present (24/26 in 2016). Table 1 shows the data and the complete analysis of the answers.

2 **Organization of medical and nursing staff in the delivery wards**

**Table 1:** Early skin-to-skin contact: practice, timing and duration

Question	2012 28 Wards	2016 26 Wards	Odds ratio (95% CI)	x^2^
STS^a^ in the delivery room is routinely offered	*N* = 27	*N* = 26	1.037 (0.4859–2.214)	*p* = 0.92
STS in the operating room is routinely offered	*N* = 11	*N* = 11	1.077 (0.3995–2.903)	*p* = 0.91
If the mother gives birth under sedation, STS is offered)	*N* = 12	*N* = 13	1.167 (0.4517–3.013)	*p* = 0.93
Within how many minutes STS begins? (0–5 minuti)	*N* = 24	*N* = 26	1.167 (0.5403–2.416)	*p* = 0.84
How long does the STS (lasts the time that the mother wants)	*N* = 18	*N* = 15	0.8974 (0.3765–2.139)	*p* = 0.98
	*N* = 5 (> 2 h)	*N* = 4	0.8615 (0.2085–3.56)	*p* = 0.87
	*N* = 2 (30 min)	*N* = 2	1.077 (0.1413–8.21)	*p* = 0.65
What position does the mother take during STS (as comfortable as possible)	*N* = 13	*N* = 7	0.5799 (0.2004–0.5799)	*p* = 0.45
What position does the newborn take during STS (prone between the mother’s breasts)	*N* = 24	*N* = 20	0.8974 (0.404–1.994)	*p* = 0.95
The cover of the newborn allows to view the face and the head	*N* = 27	*N* = 26	1.037 (0.4859–2.214)	*p* = 0.92
There are no trusted persons of the woman (father or others) during the skin to skin	*N* = 5	*N* = 2	0.4368 (0.0768–2.416)	*p* = 0.56

^a^STS = Skin to skin

In 2012 parents were informed about skin-to-skin contact prior to delivery in 24/28 (24/26) delivery wards, but not at all in the others. Parents were told which of the neonate’s vital signs (colorful and breath) to observe during early skin-to- skin contact and were instructed when to call medical staff in 10/28 delivery wards (19/26 in 2016); the rest were not informed. Routine newborn care was given in different time slots: from 3 hours to 1–2 min. Medical staff were continuously present in 15 of the wards in 2012 and in 16 in 2016, and intermittently in the remaining 13 (10/26 in 2016): with a frequency that ranged from 5 to 30 min. The neonates’ vital signs were monitored in all of the delivery wards: at non-specified intervals in 11 of them (8/26 in 2016); every 5–30 min in 16 (18/26 in 2016); and continuously in 1 ward (0/26 in 2016). Neonates’ monitored vital signs were recorded on a clinical chart in 9 delivery wards (13/26 in 2016). Table 2 shows the data and the complete analysis of the answers.

3 **Set-up of delivery and post-partum room**

**Table 2:** Organization of medical and nursing staff in the delivery wards

	2012 28 Wards	2016 26 Wards	Odds ratio (95% CI)	x^2^
Parents receive information on STS^a^	*N* = 24	*N* = 28	1.077 (0.4947–2344)	*p* = 0.99
Parents are instructed to check the vital signs (color and breathing)	*N* = 10	*N* = 19	2.046(0.8046–5.203)	*p* = 0.19
Within what the routine newborn care is performed	*N* = 8 (3 h)	*N* = 7 (3 h)	0.9423 (0.2995–2965)	*p* = 0.84
	*N* = 12 (2 h)	*N* = 7 (2 h)	0.6282 (0.2146–1.839)	*p* = 0.55
	*N* = 2 (1 h)	*N* = 4 (1 h)	2.154 (0.3635–12.76)	*p* = 0.66
	*N* = 3 (30 min)	*N* = 2(30 min)	0.7179(0.111–4.644)	*p* = 0.90
	*N* = 1 (10 min)	*N* = 0 (10 min)	/	/
	*N* = 1 (1–2 min)	*N* = 0 (1–2 min)	/	/
	*N* = 1 (at any time)	*N* = 6 (at any time)	6.462 (0.7281–57.34)	*p* = 0.06
The medical staff is present	*N* = 15 continuously	*N* = 16	1.149 (0.4747–2.78)	*p* = 0.93
	*N* = 13 discontinuous	*N* = 10	0.8284 (0.3103–2.212)	*p* = 0.89
The monitoring of vital signs by the staff every time it is done? 5–30 min	*N* = 16	*N* = 18	1.212 (0.513–2.861)	*p* = 0.82
Is it reported in the medical chart? Yes	*N* = 9	*N* = 13	1.556 (0.5702–4.856)	*p* = 0.54

^a^STS skin to skin

In 2012, all 28 delivery wards had a system in place to call for assistance; in 25 of them there was an emergency phone or bell (26/26 in 2016); in 3 other delivery wards medical staff was always nearby and available (0/26 in 2016). Lighting in the delivery and post-partum rooms was found weak in 13 delivery wards (6/26 in 2016), and only just sufficient to see the neonate’s vital signs in the remaining 15 (20/26 in 2016). Codified and written procedures were posted in 12 delivery wards (10/26 in 2016) but not in the remaining wards. In both 2012 and 2016 an emergency trolley was always at hand in all of the delivery wards surveyed. Table 3 shows the data and the complete analysis of the answers.

**Table 3:** Set-up of delivery and post-partum room

	2012 28 Wards	2016 26 Wards	Odds ratio (95% CI)	x^2^
Telephone as an emergency call tool	*N* = 25	*N* = 26	1.12 (0.5208–2.583)	*p* = 0.92
The light allows to detect the vital signs	*N* = 15	*N* = 20	1.436 (0–6099-3.38)	*p* = 0.54
Written procedure for skin to skin	*N* = 12	*N* = 10	0.8974 (0.332–2.426)	*p* = 0.96

## Discussion

The benefits and effects of skin to skin contact and the initial breastfeeding latch, immediately after birth, are widely known: increased rate and duration of breast feeding, maintenance of the neonate’s blood glucose and temperature, cardio-respiratory stability in preterm neonates, improved maternal attachment behavior and reduced crying [8, 11, 15, 17, 19]. Furthermore, it is a practice recommended by the American Academy of Pediatrics, the American College of Obstetricians and Gynecologists, the Center for Disease Control and Prevention, the Academy of Breastfeeding Medicine as well as being promoted by Unicef and its Baby Friendly Initiative [1–3, 6, 22]. Yet, at present, skin-to-skin contact is not widely adopted in delivery wards, the main reason being a lack of knowledge and education about contact at birth, and the absence of standardized guidelines for skin-to-skin contact [15–17]. Moreover, the association between early skin-to-skin contact and what is known as Sudden Unexpected Postnatal Collapse has been frequently reported and clearly described in literature on the subject [5, 11, 12, 16, 17, 19, 20]. Sudden Unexpected Postnatal Collapse (SUPC) of presumably healthy infants, within a few hours or days from birth, is a rare but recognized event. It includes any condition resulting in cardio-respiratory failure or permanent cessation of breathing [4, 5, 7, 10, 11]. The definition of SUPC varies, depending on the population studied and the authors of the publication [9]. According to the British Association of Perinatal Medicine, SUPC includes any apparently healthy neonate at, or near term (> 35 weeks gestation), who is well at birth (with a normal 5 min Apgar score and deemed able to support routine postnatal care) or considered well enough for skin-to-skin contact and routine postnatal care, suddenly collapses, i.e. is discovered in a state of cardio-respiratory extremis, such that resuscitation with intermittent positive pressure ventilation is required; or the newborn collapses within the first 7 days of life and either dies, or requires intensive care, or develops encephalopathy [21]. Although rare, SUPC can have serious consequences: in Herlenius and Kuhn’s review, both neurologic sequelae in survivors, as well as mortality, are reported as high as 50% [14]. The estimated incidence of SUPC in the first hours to days of life also varies greatly, due to differing definitions, the inclusion and exclusion of criteria, and the lack of standardized reporting; however it is considered to be 2.6–133 cases per 100,000 newborns [10, 14]. Many risk factors have already been identified and thoroughly discussed in literature, among them are primiparity; unattended skin-to-skin contact and initial breastfeeding; mother distracted by avoidable circumstances (i.e. messaging, phoning); prone and side position of the neonate; mother’s lack or little experience in breastfeeding; and inability to identify changes in the neonate’s appearance and behavior [5, 7–9, 13, 14, 18]. All of these, in different ways, influence the outcome of skin-to-skin contact and may be responsible, wholly or in part, for SUPC. In particular, early skin-to-skin contact in the first 2 hours of life coincides with the first two periods of the transitional phase from fetal to extra uterine life: the first period of reactivity, which covers the first 30 minutes of life, and the period of decreased responsiveness to stimuli, which lasts an hour and a half, followed by a second period of reactivity [14]. It is during these 90 min of decreased responsiveness that the risk factors for SUPC occurring during early skin-to-skin contact are at their highest and most dangerous. Diagnosis of SUPC requires ruling out other potential medical conditions, e.g. sepsis, cardiac disease and metabolic disease [9]. Our study clearly describes the organizational aspects of the delivery wards when a birth takes place and highlights the following: a lack of standardization of the timing and duration of early skin-to-skin contact and of the way it is performed; a lack of organization of the single delivery wards (no standard timing of routine newborn care, and recording the monitoring of vital signs during early skin-to-skin contact on the clinical chart in only 9/28 wards in 2012, increased to 13/26 in 2016, but still not a significant practice); codified and written procedures of skin-to-skin contact (present only in 12/28 delivery wards in 2012 and in 10/26 in 2016); a lack of counseling on the potential risks of unsafe positions of both mother and baby during early skin-to-skin contact; a lack of continuous observation of mother-baby dyad during early skin-to-skin contact (in nearly half of the delivery wards); and a potential safety issue concerning weak lighting (in 13/28 wards in 2012 and in 6/26 in 2016, while only just sufficient to see the neonate’s vital signs in the remaining wards, a very slight improvement). Yet what also emerges from this survey is the strong effort made to promote and implement early skin-to-skin contact: in all the delivery wards after a natural birth, in 11 out of 28 wards after caesarean sections in 2012, 11/26 in 2016; and suggested to mothers who have given birth under sedation in 12/28 delivery wards in 2012 and 13/26 in 2016 - not a significant improvement however. Moreover, the skin-to-skin operation in the operating room requires the presence of dedicated personnel to assist the mother-child dyad. The monitoring of the mother-infant dyad is therefore very close, and consequently also the prevention of SUPC. To date, research on SUPC has focused mainly on the definition, etiology, incidence and clinical aspects of SUPC. Our study focuses instead on the practical and organizational aspects of the delivery wards that may lie behind the occurrence SUPC, highlighting on one hand the importance of first carefully and thoroughly assessing all those aspects that must be addressed and modified to minimize, as much as possible, the risk of SUPC; on the other hand the necessity of then standardizing the timing and duration, and the methods and procedures of early skin-to-skin contact. Attempts to standardize methods and procedures aimed at providing safe post-partum transition, and safe, immediate and uninterrupted skin-to-skin contact with continued monitoring of mother and neonate have already been made. However, none of these have yet been proven to concretely reduce the risk of sentinel events and SUPC. The AAP has recently collected and organized all available suggestions supporting the safe implementation of skin-to-skin contact, together with rooming-in guidelines [9]. Our longitudinal survey (2012 vs. 2016) reveals that although there are no certain indications for preventing SUPC, the individual delivery wards tend to spontaneously improve assistance, based on the international literature available, although not always in significant measures (for example, skin-to-skin contact in the operating theatre; the constant presence of hospital staff; sufficient illumination in the room for the correct observation of the newborn, etc.). Other aspects need to be specifically addressed and important modifications made (i.e. availability or posting of written procedures; suggesting safer positions to mothers, etc.). In light of all that emerged from our survey, we hereby offer the following recommendations, the efficacy of which we intend to evaluate after having been applied for a pre-determined period of time [23–27]:

- Complete, clear and detailed information on the advantages of skin-to-skin contact, as well as on the associated risks and safety concerns, should be always given, both orally and written, to mother and partner before birth takes place.
- All efforts should be made to assure continuous monitoring of mother and neonate during early skin-to-skin contact, and even more so if the mother is primiparus, exhausted or has been sedated.
- As soon as the mother is admitted, parents should be invited to temporarily give up the use of mobile phones, or any other electronic gadget which might cause distraction, leaving them with staff during birth and early skin-to-skin contact [18].
- Technical details such as room lighting or calling for help systems, should be assessed periodically and renewed or replaced when necessary.

- Non-routine monitoring with pulse oximetry, if not in particular conditions such as absence of family member, language barrier, excessive workload, reduced personnel [28].

In 2017, the Department of Health of the Piedmont Region in collaboration with that of the Valle d’Aosta Region organized an update day for all the directors and the heads of the birth points of the 2 regions in order to report the surveillance data carried out in the 2012 and 2016, to reaffirm the good practices related to the birth event and to share systematic improvement actions. In all 28 of the delivery wards surveyed in Piedmont and the Aosta Valley, a new protocol on the physiological approach to newborns is currently in progress: another survey will be carried out during the course of next year (2020) to assess changes after its application.

In all 28 of the delivery wards surveyed in Piedmont and the Aosta Valley, a new protocol on the physiological approach to newborns is currently in progress: another survey will be carried out during the course of next year (2020) to assess changes after its application.

The survey carried out has some methodological limits, in fact the answers regarding the timing of the start of skin-to-skin contact and the consequent duration, despite the survey asked to specify the average interval in minutes from the beginning of the contact and duration, offering three response options (at least 30 min, at least 1 h and at least 2 h), in the absence of objective data can only represent the general impression of the people who completed the questionnaire. Furthermore the causes of suspension indicated with “on obstetric indication” or “on pediatric indication”, were not specified by the participants in the survey. Unfortunately in Italy and in the Piedmont and Valle d’Aosta regions there is no register for SUPC reports, the cases identified have been reported as anecdotal cases. Furthermore, the description of practice in the delivery rooms is linked to the answers to a questionnaire and there has been no description by external observers who have capillaryly observed the habits of each birth point “live”.

## Conclusions

The importance of early skin-to-skin contact after birth, as well as the inherent risk factors that may cause Sudden Unexpected Postnatal Collapse, a rare but yet recognized event, led to the necessity of evaluating how best to reduce the risk of its occurrence. The first step taken was a careful examination and identification of the environmental and logistical aspects needing to be addressed and eventually changed in the delivery wards. The longitudinal evaluation (2012 vs. 2016) did not reveal significant changes, either because some good care practices had already been consolidated in the 2012 evaluation (for example immediate skin-to-skin in the delivery room), and because in most wards there were no written or codified procedures; or because no significant changes had been made in the assistance given; or where some improvements had been recorded, they were not however statistically significant. The interest in performing skin to skin is very high, but in our sample only two hospitals are BFH certified while two others are in the certification phase. Therefore, specific indications are proposed based on experiences reported in the literature, and on the opinion of the authors (Table 4) that will be soon be evaluated in a new survey. We believe that conducting surveys at regular intervals, either in individual delivery wards, or better yet, in a cluster of wards, will lead to a better evaluation of where changes need to be made, as well as promote the positive results achieved, so that safe and successful early skin-to-skin care can eventually be implemented in all birth centres.

**Table 4:** Raccomendations and preventive measures for safe postpartum mother-child skin-to-skin contact

Furnish a written protocol regarding assistance, observation/supervision and of parent instruction	
Maintain sufficient lighting for the correct observation of the newborn	
Never leave the mother alone, always ensure the presence of the hospital staff, relatives or a trusted person (sensitized prior to delivery)	
Favor optimal 45° sitting position of the mother (biological nurturing): this guarantees the use of active neonatal reflexes that promote breastfeeding, neonatal respiration, and greater interaction in the mother-infand dyad, including eye contact	
Ensure that infant’s mouth and nose are always visible	
Ensure continuous supervision by staff during skin-to-skin contact in cases of maternal sedation, fatigue or primiparity	
Avoid use of mobile phones and other distractions in the room	
Use pulse oximetry monitoring in highly selected situations	

Adapted from Davanzo R, et al.; Making the first days of life safer: preventing sudden unexpected postnatal collapse while promoting breastfeeding. J Hum Lact 2015 31: 47–52 [9]

## Additional file

Additional file 1: Questionnaire in sections used for the survey. (DOC 26 kb)

## Data Availability

Not applicable.

## Abbreviations

SUPC: Sudden Unexpected Postnatal Collapse
