# Supplementary material for: Skin-to-skin contact and delivery room practices: a longitudinal survey conducted in Piedmont and the Aosta Valley
Source: Ital J Pediatr. 2019 Aug 2;45:95. doi: 10.1186/s13052-019-0688-9 (PMC6679473; doi:10.1186/s13052-019-0688-9)
Supplement: Supplementary file 1 — Questionnaire in sections used for the survey. (DOC 26 kb) [file 13052_2019_688_MOESM1_ESM.doc]

| 1 - Post-Partum Skin to Skin in delivery room after normal child birth does Skin to Skin between mother and newborn normally take place? | | YES  NO |
| --- | --- | --- |
| □ Straight away in the after delivery ward  □ Shortly after in the after delivery ward  □ After in the maternity ward | | |
| 2 - Specify how many minutes on average Skin to Skin contact starts after delivery | | YES  NO |
| 3 - How long does Skin to Skin last ?  □ At least 30 minutes  □ At least 1 hour  □ At least 2 hours  □ As long as the mother wants  □ Other reasons. To specify______________________________ | | |
| 4 - How is the mother usually positioned ?  □ Lying on her back  □ Lying on her side  □ Sitting  □ Half- sitting  □ As comfortably as possible  □ Anyway | | |
| 5 - How is the neonate positioned  □ The neonate is prone on the mother’s breast  □ Lying supine on the mother’s side  □ Lying on one side, on the mother’s side  □ On the mother’s side in the mother’s arm’s hollow | | |
| 6 - The neonate is  □ Completely covered, not visible  □ Covered, but with its head visible | | |
| 7 - How is the light in the room ?  □ Suffused  □ Just enough to see neonate’s vital signs  □ Full on | | |
| 8 - Is non -medical staff present in the room where Skin to Skin takes place?  Who? _____________________________________ | YES  NO | |
| 9 - Is medical staff present in the room where Skin to Skin takes place?  □ Always present  □ Not always present Every how many minutes?_____________________ | YES  NO | |
| 10 - Are neonate’s vital signs monitored by the medical staff during Skin to Skin?  How often?_______________________________ | YES  NO | |
| 11 - Are the monitored vital signs reported in the clinical chart? | YES  NO | |
| 12 - In case of emergency is there a way to ask for help?  If so, how?_____________________________ | YES  NO | |
| 13 - Is an emergency trolley available in the near vicinity? | YES  NO | |
| 14 - Are parents informed about Skin to Skin contact before delivery? | YES  NO | |
| 15 - Are parents informed about which vital signs of the neonate to observe? | YES  NO | |
| 16 - Are parents told when, if necessary, to call the medical staff? | YES  NO | |
| 17 - Is there a written procedure for Skin to Skin contact? | YES  NO | |
| 18 - If the mother, who has just given birth, is under sedation is Skin to Skin contact suggested ? | YES  NO | |
| 19 - Are special precautions taken?  Which?_________________________________ | YES  NO | |
| 20 - Does Skin to Skin take place in the operating theatre after Caesarean section? | YES  NO | |
| 21 - How long after birth does the neonate receive primary neonatal care (washing, Vitamin K, eye drops )? _________________________________________ | | |
